# The Effects of Stationary Phases on Retention and Selectivity of Oligonucleotides in IP-RP-HPLC

**DOI:** 10.1007/s10337-014-2766-x

**Published:** 2014-09-16

**Authors:** Sylwia Studzińska, Lidia Pietrzak, Bogusław Buszewski

**Affiliations:** Chair of the Environmental Chemistry and Bioanalytics, Faculty of Chemistry, Nicolaus Copernicus University, 7 Gagarin St, PL-87 100 Torun, Poland

**Keywords:** Ion-pair liquid chromatography, Ion-pair reagents, Oligonucleotides, Retention mechanism, Selective stationary phases

## Abstract

There is a growing demand for the separation and identification of short nucleic acid fragments, such as oligonucleotides. There were two main goals of the present investigation, namely, evaluation of the impact of stationary phase type and the influence of various ion-pair reagents on the retention behavior of oligonucleotides in ion-pair liquid chromatography. Three types of ion-pair reagents were studied: triethylammonium acetate, dimethylbuthylammonium acetate and mixtures of 1,1,1,3,3,3-hexafluoro-2-propanol and triethylamine. Two novel types of packing materials, namely, cholesterol and alkylamide were used for this purpose for the first time. The results indicate that the mechanism of oligonucleotides retention is determined by the hydrophobicity of ion-pair reagents and polar ligands localized on the surface of stationary phases. Oligonucleotides were most effectively separated with the use of alkylamide and cholesterol packings. These two stationary phases reduce the time of analysis in comparison with the octadecyl packing material. Moreover, separation was achieved under non-denaturating conditions.

## Introduction

Oligonucleotides are short (3–200 nucleotides), single-stranded fragments of nucleic acids. They are built of nucleotide mers that are composed of a purine or pyrimidine base, pentose sugar and phosphate residues [1]. Natural oligonucleotides exist in the form of small RNA molecules or intermediates derived from the breakdown of large nucleic acids [2]. Oligonucleotides may be also chemically synthesized and used in various fields of science, including as primers for DNA amplification in the polymerase chain reaction (PCR) or as therapeutics against cancer and viral infection [3–6]. The critical requirement for these compounds is their high purity. Quality control of synthetic oligonucleotides, determination of shorter ones and related compounds is usually accomplished by chromatographic techniques, especially ion-pair high-performance liquid chromatography (IP-RP-HPLC) [7–15]. The separation of oligonucleotide depends on two mechanisms: hydrophobic interaction and charge-to-charge interaction between negatively charged oligonucleotide and positively charged alkylammonium ions in mobile phase. Weak IP systems make the first interaction dominant, while strong IP systems convert the separation nearly completely to charge-based separation with minimal effect of nucleobases hydrophobicity [16].

Triethylamine with acetic acid are commonly used as the counter ion in oligonucleotides analysis [9, 10]. However, a number of studies have introduced the use of other alkylammonium salts and there is a growing interest in new IP reagents, which will improve separation efficiency of oligonucleotides [17–19]. More recently, Levin et al. [19] published an extensive and relevant IP-RP-HPLC oligonucleotide study concerning the development of multiple ion-pairing agents combined in the mobile phase. It was proven that they can improve the overall chromatographic resolution and peak shape of studied analytes in comparison with the use of a single ion-pair (IP) reagent alone [19]. The study of Apffel et al. [15] is of great importance, because he introduced volatile triethylamine with 1,1,1,3,3,3-hexafluoro-2-propanol as an IP reagent for the analysis of nucleic acid components. This novel additive resulted in good HPLC separation and efficient negative ion mode electrospray ionization. The authors observed small suppression of mass spectrometry signal suppression. Moreover, they achieved acceptable separation of native and synthetic oligonucleotides [15]. Since its introduction, the HFIP/TEA buffer has become the most widely used for liquid chromatography mass spectrometry (LC-MS) determination of these compounds [20, 21].

An octadecyl stationary phase was used in case of most of investigations focusing on the determination of oligonucleotides [15–21]. A rare but noteworthy are attempts to determine the suitability of mixed-mode columns. An example is work of Biba et al. [23] who evaluated several commercial columns consisting of ion-exchange and octadecyl ligands for RNA oligonucleotide analysis. The structural and positional isomers of studied biomolecules were separated due to the properties of these stationary phases, when typical anion-exchange mobile phases were used [23].

In this study, the retention and separation of eight semi-complementary oligonucleotides was analyzed under non-denaturing conditions in the IP RP LC mode. There were two main goals of investigation, namely: evaluation of the impact of specific and new stationary phase type, and the impact of various modifiers (ion-pair reagents) of mobile phase on the retention behavior of oligonucleotides. Packing materials with hydrophobic and hydrophilic moieties bonded to a silica support were selected for this purpose. Moreover, three IP reagents were used: triethylammonium acetate (TEAA), dimethylbuthylammonium acetate (DMBAA) and mixtures of 1,1,1,3,3,3-hexafluor-2-propanol and triethylamine (HFIP/TEA). The best chromatographic conditions for oligonucleotide separation were selected.

## Materials and Methods

### Oligonucleotides Samples and Chemicals

Oligonucleotide standards were purchased from GeneSys (Wrocław, Poland). The sequences of the analyzed compounds are presented in Table 1. Oligonucleotides were supplied in lyophilized form and standard solutions were prepared by dissolution in deionized water. The analyzed compounds had the concentration of 0.1 mM.

**Table 1:** The basic characteristics of oligonucleotides used in the investigations

Shortcut	Sequence of oligonucleotides (5′–3′)	Molecular weight (g mol^−1^)	Melting temperature (°C)	Percentage part of bases
OL1	ATC GAT CGA TCG ATC GAT CG	6,113	45.4	25 %A, 25 %T, 25 %C, 25 %G
OL2	ATC GAT CGA TCG ATC GAT C**A**	6,098	43.4	30 %A, 25 %T, 25 %C, 20 %G
OL3	ATC GAT CGA **A**CG ATC GAT CG	6,122	45.4	30 %A, 20 %T, 25 %C, 25 %G
OL4	ATC GAT CGA T**A**G ATC GAT CG	6,137	43.4	30 %A, 25 %T, 20 %C, 25 %G
OL5	ATC GAT CGA TCG ATC GAT C**C**	6,074	45.4	25 %A, 25 %T, 30 %C, 20 %G
OL6	ATC GAT CGA TCG ATC GA**A** CG	6,122	45.4	30 %A, 20 %T, 25 %C, 25 %G
OL7	ATC GAT CGA **G**CG ATC GAT CG	6,147	47.5	25 %A, 20 %T, 25 %C, 30 %G
OL8	ATC GA**A** CGA TCG ATC GAT CG	6,122	45.4	30 %A, 20 %T, 25 %C, 25 %G

Mobile phases were prepared with the use of organic solvents, including methanol and gradient grade acetonitrile (J.T. Baker, Deventer, Holland). Mobile phases were modified with high purity ion-pair reagents: TEAA buffer, 1,1,1,3,3,3-hexafluoro-2-propanol, *N*,*N*-dimethylbutylamine acetate and triethylamine (Sigma-Aldrich, Dorset, UK). Deionized water was obtained from the Milli-Q system (Millipore, El Passo, TX, USA).

### HPLC Instrumentation and IPC Conditions

The UltiMate^®^ 3000 Binary Rapid Separation LC (RSLC) (Dionex, Sunnyvale, CA, USA) ultra high-performance liquid chromatography (UHPLC) system equipped with a diode-array detector was used in the study. Data were collected with the use of Thermo Scientific Dionex Chromeleon 7 Chromatography Data System program.

Three stationary phases were used: octadecyl (SG-C18), cholesterol (SG-CHOL) and alkylamide (SG-AP) (Table 2). They were prepared in our laboratory according to a previously described synthesis method [24–26]. Column dimensions and the carbon load on the stationary phase are presented in Table 2. All stationary phases were prepared from the same batch of 5 μm Kromasil^®^ silica gel with 300 Å pore volume. The stationary phase was packed into stainless-steel columns with the use of home-made apparatus equipped with a Haskel pump (Burbank, CA, USA) under constant pressure. Column void volume (*t*_0_) was measured by injecting methanol. Other detailed chromatographic conditions were listed in the figure caption.

**Table 2:** Characteristic of stationary phases used in the investigation

Stationary phase	Column dimensions (mm)	Modification stage					Functional group	Types of possible interactsions
		Carbon load (%)			Coverage density (μmol/m^2^)			
		Ist	IInd		Ist	IInd		
	125 × 2.0	7.90	–		3.36	–	Hydroxyl octadecyl ligands	Hydrogen bonds Hydrophobic
	125 × 4.6	1.35	5.17		3.59	3.49	Hydroxyl amide group Aminopropyl alkyl chains	Hydrogen bonds Hydrogen bonds Donor–acceptor Hydrophobic
	125 × 4.6	1.35	8.62		3.59	2.61	Hydroxyl Amide group Aminopropyl Double bond Alkyl chains Steroid rings	Hydrogen bonds Hydrogen bonds Donor–acceptor π…π type Hydrophobic London Dispersion Forces

TEAA solutions were prepared by the dilution of 1 M of preformulated commercial buffer. Four different concentrations of TEAA were studied: 25, 50, 75 and 100 mM. DMBAA was prepared by the addition of proper volume of ion-pair reagent to water (pH was between 10 and 12) and adjusting the pH to 6.8–7.0 with the glacial acetic acid. The 5, 10, 15 and 20 mM of DMBAA were tested during the investigation. The HFIP/TEA was prepared in two steps by titrating acid solution (HFIP) with TEA. Firstly, the stock solution of 400 mM HFIP in water was prepared. Next TEA was slowly added to the solution. The pH of HFIP/TEA was in range of 6.7–7. Concentrations of TEA were equal to 2.54, 2.96, 3.39 and 3.80 mM.

## Results and Discussion

### Stationary Phase Selection

The properties and retention mechanism of octadecyl (SG-C18) have been extensively described in the literature [18, 26]. There are very little attempts to involve other packing materials for the investigation of oligonucleotides, although this direction seems to be very interesting. The main aim of the experiment was to evaluate the applicability of two other types of stationary phases in the oligonucleotide analysis. Packing materials containing hydrophobic and hydrophilic moieties bonded to a silica support have been used: alkylamide (SG-AP) and cholesterol (SG-CHOL) stationary phases (Table 2). They were never used in the analysis of oligonucleotides before. Both of them were synthesized on the basis of the same aminopropyl support, therefore, they contain residual aminopropyl groups on the surface. In the second step of synthesis process, alkylamide groups are chemically bonded to the SG-AP surface, while cholesterol molecule is attached in case of SG-CHOL. Table 2 presents the percentage part of carbon on the stationary phases together with types of functional groups. The alkyl chains are localized at SG-C18 surface with 7.9 % of carbon, while SG-AP has the lowest carbon content on the support surface, equal 5.17 % (Table 2). The surface of the SG-CHOL stationary phase was modified with large nonpolar cholesterol molecule, consequently it poses the highest carbon content (Table 2). Although both packing materials pose aminopropyl groups, it should be clearly noted that they are not protonated under IP-RP-HPLC conditions. Therefore, these packing materials were not used as anion-exchangers during present investigations. The use of mixed-mode columns has its own challenges, because all possible interactions between ligands and different functional groups of the oligonucleotide must be considered, and they may create unpredictable or undesirable results [25]. For this reason, systematic studies were performed during the investigations. On the other hand we have expected that decreasing the polarity of stationary phases used for the chromatographic analysis of oligonucleotides will cause reduction of time needed for their separation and will improve the selectivity. Therefore, these two columns were selected for present investigation.

Oligonucleotides with varying the motif and sequence composition while maintaining the length of compounds were selected (Table 1). The alteration or substitution of one nucleotide in the sequence is meant to mimic synthetic impurities, therefore these compounds are important class of possible contamination. All of the tested oligonucleotides are self-complementary molecules, which forms hairpin loops. Furthermore, they were chromatographically analyzed under non-denaturing conditions at a relatively low temperature of 30 °C and neutral pH. These conditions were chosen to prove utility of studied stationary phases in the separation of oligonucleotides, despite their secondary structure.

### Mobile Phase Selection

One of the main goals of research was the optimization of mobile phase conditions for the separation of oligonucleotides with the use of selected stationary phases. Three buffer systems were chosen for this purpose. TEAA buffer is most commonly used in oligonucleotide analysis. DMBAA has the same molecular formula and similar physicochemical properties, but its structure is different. Both TEA and DMBA are strong base, the pK_a_ values are equal 10.7 and 10.19 respectively. They are active ion-pairing agents during the chromatographic process. HFIP is more volatile (b.p. 59 °C) and at pH 7–8.3 is only partially ionized (pK_a_ 8.25) in comparison with acetic acid (pKa = 4.75, bp = 118 °C). The mixtures of TEA and HFIP were also used. HFIP is a very weak acid, it has a high ability to form hydrogen bonds and can interact and mix with most acceptor solvents. Moreover, it is not charged during the chromatographic run and can be freely evaporated. HFIP is added to the aqueous-organic mobile phase to decrease the solubility of TEA, consequently its distribution between the mobile and stationary phase is changing.

In the first step of the analysis, the type and percentage part of organic solvent in the eluent were estimated. Acetonitrile was used for TEAA and DMBAA due to its greater elution strength in comparison with methanol. Methanol was used for HFIP/TEA, because HFIP is insoluble in acetonitrile. The percentage parts of acetonitrile and methanol were determined based on the results of several chromatographic analyses. For SG-C18 and SG-AP 10 % (v/v) ACN (TEAA, DMBAA) and 20 % (v/v) MeOH (HFIP/TEA) were chosen, while for SG-CHOL, the best results were noted for 15 % (v/v) ACN and 30 % (v/v) MeOH.

### The Impact of Ion-Pair Reagent Concentrations

The impact of IP reagent concentration on the oligonucleotide retention factor *k* was examined. The data in Table 3 demonstrates the influence of the type and concentrations of IP reagents on oligonucleotide retention for all tested columns. An increase in *k* values with an increase in the concentration of alkylammonium ions in the mobile phase were reported for all tested columns. The greatest differences in retention times of the tested oligonucleotides were noted at the highest concentrations of TEAA, DMBAA and HFIP/TEA. We have demonstrated that although DMBAA concentrations were fivefold lower than TEAA, *k* values for oligonucleotides were significantly higher (Table 3). The above can be attributed to the molecular structure of amine: DMBA cation comprises an *n*-alkyl chain of four atoms, whereas TEA^+^ possesses only ethyl groups. Consequently, a DMBAA IP reagent is strongly adsorbed on the surface of the stationary phase due to stronger hydrophobic interactions between the alkyl chain of the IP reagent and the surface of packing material. Therefore, a dynamically generated charge appears on the modified surface of the support. IP reagents then electrostatically interact with negatively charged oligonucleotides to create ion-pairs in the mobile phase.

**Table 3:** The retention factor *k* values of oligonucleotides for all stationary phases and ion-pair reagents used in the study

Oligonucleotide	*k*										
	TEAA				DMBAA				TEA/HFIP		
	25 mM	50 mM	75 mM	100 mM	5 mM	10 mM	15 mM	20 mM	2.54 mM	2.96 mM	3.39 mM
SG-C18											
OL 1	0.04	0.31	0.50	0.69	0.35	4.80	10.51	21.32	0.75	1.46	3.83
OL 2	0.11	0.63	1.06	1.51	0.72	8.26	22.19	44.74	0.58	2.17	5.22
OL 3	0.03	0.34	0.58	0.81	0.35	5.48	13.95	27.14	0.28	2.07	3.91
OL 4	0.04	0.36	0.60	0.86	0.34	6.00	14.92	30.14	0.43	2.36	4.58
OL 5	0.08	1.16	1.50	1.62	0.43	3.85	15.14	30.59	0.38	2.45	4.49
OL 6	0.12	0.67	1.64	1.91	0.64	4.58	17.74	35.45	0.38	2.50	4.32
OL 7	0.03	0.39	0.84	0.97	0.29	5.59	10.37	20.03	0.23	1.80	3.04
OL 8	0.07	0.53	1.22	1.49	0.54	3.85	10.80	30.26	0.38	2.60	4.15
OL 1	0.14	0.36	0.71	0.83	0.21	0.53	1.72	3.37	~t_0_	0.57	0.66
SG-AP											
OL 2	0.27	0.67	1.25	1.51	0.27	0.70	2.99	5.86	0.16	0.70	0.51
OL 3	0.16	0.43	0.81	1.00	0.16	0.44	2.10	4.19	~t_0_	0.51	0.64
OL 4	0.17	0.45	0.86	1.05	0.28	0.50	2.24	4.50	0.01	0.50	0.84
OL 5	0.15	0.46	0.86	1.08	0.37	0.50	2.11	4.42	0.11	0.57	0.80
OL 6	0.20	0.50	0.91	1.16	0.37	0.66	2.43	4.81	0.04	0.58	0.82
OL 7	0.10	0.30	0.57	0.70	0.21	0.34	1.43	2.87	~t_0_	0.35	0.51
OL 8	0.16	0.39	0.69	0.87	0.58	0.52	1.90	3.79	0.01	0.53	1.03
SG-CHOL											
OL 1	~t_0_	0.16	0.28	0.38	~t_0_	0.03	0.30	0.58	–	–	–
OL 2	~t_0_	0.27	0.54	0.72	~t_0_	0.07	0.50	0.81	–	–	–
OL 3	~t_0_	0.15	0.33	0.44	~t_0_	0.03	0.38	0.63	–	–	–
OL 4	~t_0_	0.17	0.33	0.46	~t_0_	0.03	0.39	0.66	–	–	–
OL 5	~t_0_	0.17	0.37	0.50	~t_0_	0.03	0.37	0.63	–	–	–
OL 6	~t_0_	0.19	0.40	0.52	~t_0_	0.04	0.41	0.67	–	–	–
OL 7	~t_0_	0.10	0.21	0.28	~t_0_	0.02	0.31	0.53	–	–	–
OL 8	~t_0_	0.12	0.27	0.34	~t_0_	0.03	0.36	1.15	–	–	–

Experimental conditions for SG-C18: 90 % (v/v) of TEAA or DMBAA and 10 % (v/v) of acetonitrile; 80 % (v/v) of HFIP/TEA and 20 % (v/v) of methanol; flow rate 0.2 mL min^−1^. For SG-AP: 90 % (v/v) of TEAA or DMBAA and 10 % (v/v) of acetonitrile; 80 % (v/v) of HFIP/TEA and 20 % (v/v) of methanol; flow rate 0.5 mL min^−1^. For SG-CHOL: 85 % (v/v) of TEAA or DMBAA and 15 % (v/v) acetonitrile; flow rate 1.0 mL min^−1^. The autosampler and column temperature 30 °C. UV–Vis detection λ = 254 nm. Injection volume 0.5 µL

Triethylamine concentrations had no impact on oligonucleotide retention for SG-CHOL; therefore, the results for HFIP/TEA are not shown in Table 3. The above can be attributed to the use of excessively low, ineffective concentrations of TEA.

Since all columns used in the study were prepared in our laboratory, the repeatability data are of great importance. The repeatability of retention times was measured by double injection of each studied oligonucleotide. It was expressed as relative standard deviation (RSD). For the SG-C18 column, RSD was always lower than 6.2 %. In case of SG-CHOL RSD was lower than 4.4 % while for SG-AP it never exceeded 4.5 %. The greatest RSD was determined in case of OL1.

### The Influence of Stationary Phase Type on Oligonucleotide Retention

The influence of stationary phase type on the retention of the analyzed oligonucleotides is presented in Table 3. A significant increase in *k* values was observed for SG-CHOL in comparison with SG-C18 (Table 3), therefore 15 % (v/v) of acetonitrile was used for cholesterol packing material. Contrary results were reported for SG-AP. The *k* values were nearly two-fold lower for most of the analyzed compounds in comparison with SG-C18, when TEAA was used (Table 3). Moreover, the oligonucleotides retention for SG-AP was sevenfold lower in comparison with SG-C18 for DMBAA (Table 3). The elution order of studied biomolecules for SG-AP was changed in comparison with SG-C18 (Table 3). The above can be attributed to different retention mechanism for SG-AP and SG-C18. In SG-AP, polar interactions between the stationary phase and oligonucleotides significantly affect the chromatographic behavior of the analyzed biomolecules.

### The Discussion on Retention Mechanism of Oligonucleotides

The results of this study indicate that the oligonucleotide retention mechanism is determined by both: the chemical structure of the IP reagent and polar groups in the stationary phase. The mixed nature of the retention mechanism supports several types of interactions during chromatographic analysis of oligonucleotides, including hydrophobic, polar and electrostatic interactions (Table 2). The structure of stationary phase has a great influence on these interactions. The lowest *k* values for oligonucleotides were determined for SG-AP (Table 3). Similarly to SG-C18, SG-AP poses alkyl chains, but they are composed of only 12 carbon atoms. Moreover, the coverage density of alkyl ligands is lower in comparison with SG-C18 (Table 2) and consequently, there is a lower probability of the adsorption of IP reagent by hydrophobic interaction. It should also be noted that the coverage density for both steps of SG-AP synthesis is similar. Consequently, during the second step of preparation of this packing, almost all of aminopropyl groups have been modified and converted to alkylamide ligands (Table 2). Accordingly, the aminopropyl groups do not affect retention of oligonucleotides. SG-CHOL was characterized by the highest *k* values (Table 3). The carbon content is greater for SG-CHOL than for SG-AP and SG-C18, because of the size of bulky cholesterol groups (Table 2). Consequently, these groups retain oligonucleotides more effectively than other ligands in SG-C18 or SG-AP (Table 3). Furthermore, due to the presence of double bonds in the structure of cholesterol molecule, π–π interactions also influence the retention of oligonucleotides (Table 2). However, a significant role in the retention mechanism on SG-CHOL play probably also aminopropyl groups. Contrary to SG-AP, in this case, only a part of aminopropyl groups have been modified and some of them were present on the surface of stationary phase. A donor–acceptor interactions between these groups and oligonucleotides are probably the cause of a high retention of the tested compounds. In summary, the highest *k* values for SG-CHOL are caused by the presence of several functional groups, which may interact with oligonucleotides by hydrophobic, polar and electrostatic interactions (Table 2).

### The Influence of Nucleobases on Oligonucleotides Retention

Retention data can be used to estimate the impact of nucleobases on *k* values of the analyzed compounds. The effect of changes in oligonucleotide sequence was evaluated based on the data presented in Table 3. Similar trends were noted for all stationary phases. The alternation of guanidine with adenine at the 10th and 20th position increased *k* values of oligonucleotides (OL7 and OL3, OL1 and OL2) (Tables 1, 3). The above could have resulted from greater hydrophobicity of the adenine base that does not contain an oxygen atom.

Several oligonucleotides where cytidine was substituted with adenine were also analyzed (OL5 and OL2, OL1 and OL4) (Table 1). In each case, such substitution increased the retention of analyzed compounds (Table 3). The same effect was observed when thymidine was replaced with adenine (OL1 and OL6, OL8 and OL3) (Table 1). The above can be attributed to the unique structure of nucleotides: adenine belongs to the group of purines, which have greater retention than pyrimidines (cytidine and thymidine).

Nevertheless, the cause of changes in retention times of sequence isomers (OL3, OL6, OL8) cannot be reliably determined without a knowledge of the secondary structure of oligonucleotides. They were analyzed under non-denaturing conditions; therefore, their secondary structure will influence the retention to a great extent. Some fragments of each oligomer sequence are complementary, what could be the reason for the intermolecular base-pairing and forming hairpin loops. In most cases (OL1, OL2, OL3, OL4, OL7) the loops will be formed between 10th and 14th nucleotide in the sequence (Table 1). Consequently, the interaction of oligonucleotide with stationary phase surface will depend mainly on the type of nucleobases, forming the loops, and on the nucleobases present at the ends of sequence.

**Fig. 1:**
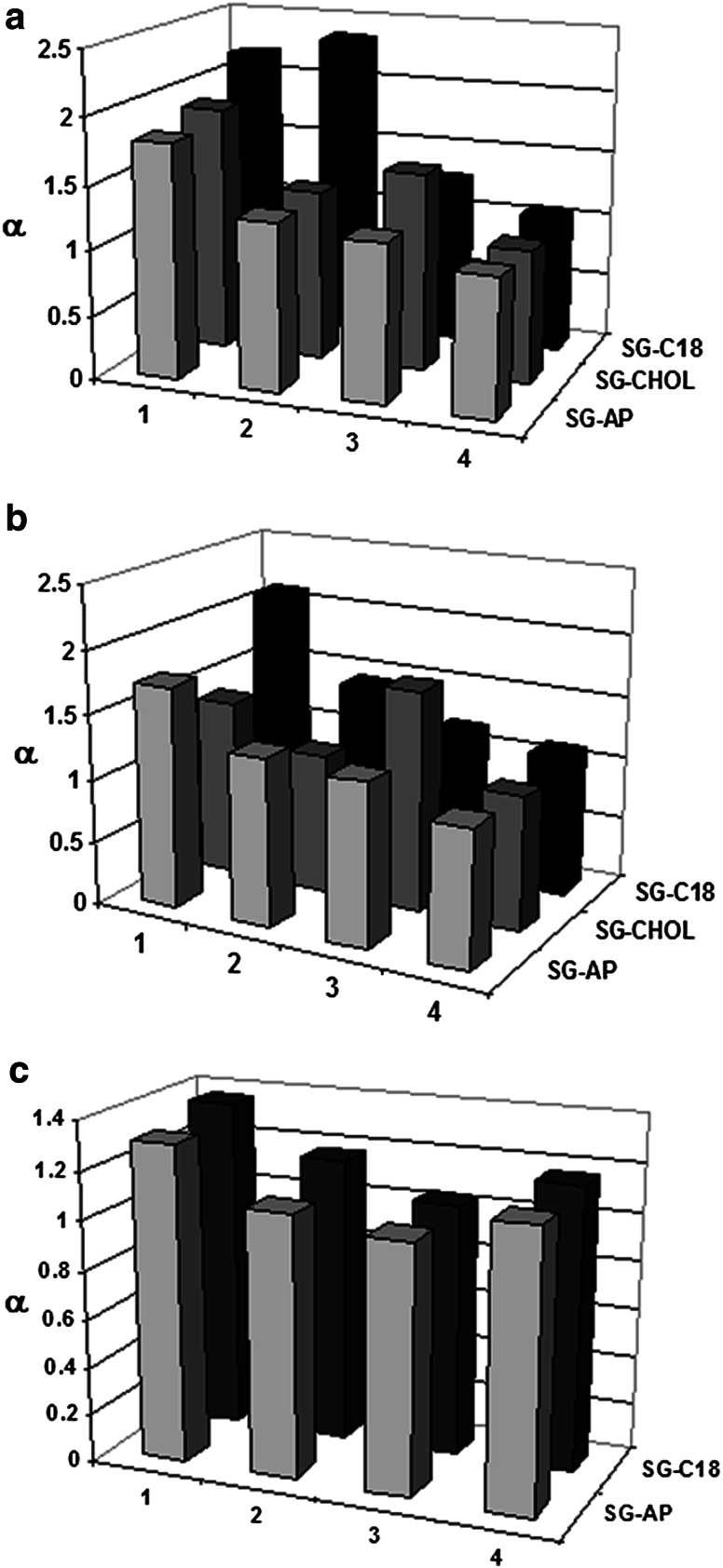
The impact of position of nucleobases in the oligonucleotide sequence on selectivity factor (α) for various stationary phases: (**a**) 100 mM of TEAA, (**b**) 20 mM DMBAA, (**c**) 3.39 mM TEA and 400 mM HFIP. Notation:* 1*—pair of OL1 and OL2;* 2*—pair of OL1 and OL5;* 3*—pair of OL6 and OL8;* 4*—pair of OL3 and OL4. For detailed chromatographic conditions see “HPLC Instrumentation and IPC Conditions” section

The impact of terminal variation in sequence (OL1, OL2, OL5) versus single nucleotide internal position change (OL6 and OL8, OL3 and OL4) was estimated on the basis of chromatographic selectivity factor (α). α was calculated the pairs of oligonucleotides: OL1 and OL2 (α_1_), OL1 and OL5 (α_2_), OL6 and OL8 (α_3_), OL3 and OL4 (α_4_). It was determined for all of stationary phases and various concentrations of IP reagents. It appeared that α had similar values independently of concentration, therefore, only the highest concentrations were selected for comparison between different IP reagents and packing materials. Next, selectivity factors were used to construct graphs presented in Fig. 1. Similar tendencies were observed for SG-C18 and SG-AP: α values were higher for pairs of oligonucleotides differing in the type of terminal nucleotides in comparison with pairs of various internal positions of nucleobases (Fig. 1; Table 1). The access of 3′- or 5′-end of oligonucleotide to the stationary phase surface is greater and easier in comparison with large hairpin loop, therefore there will be greater retention differences between analytes of various nucleotide positions at terminal positions of sequence.

Opposite effect was noticed for SG-CHOL for which α_3_ had higher values than α_2_ or even α_3_ (Fig. 1a, b). It proves different selectivity of SG-CHOL in comparison with SG-AP or SG-C18. This packing material poses bulky cholesterol molecule bonded to aminopropyl ligands, consequently the access of analyzed biomolecules to the surface of support is different. For this reason, the differences of α between studied pairs of biomolecules are not influenced by changes in the terminal or internal positions of oligonucleotides in the sequence. Moreover, SG-CHOL interacts with analyzed compounds with different types of interactions compared to SG-C18 and SG-AP, as it was already summarized in "The Discussion on Retention Mechanism of Oligonucleotides" section.

Figure 1c presents results obtained for HFIP/TEA mobile phase. α values are similar for SG-AP and SG-C18 and also for pairs of oligonucleotides. This tendency proves effect observed earlier in the literature: mobile phases containing HFIP/TEA have lower separation efficiency in comparison with other solvents used in IP-RP-HPLC for the analysis of these biomolecules [19–22].

### Oligonucleotide Separation

**Fig. 2:**
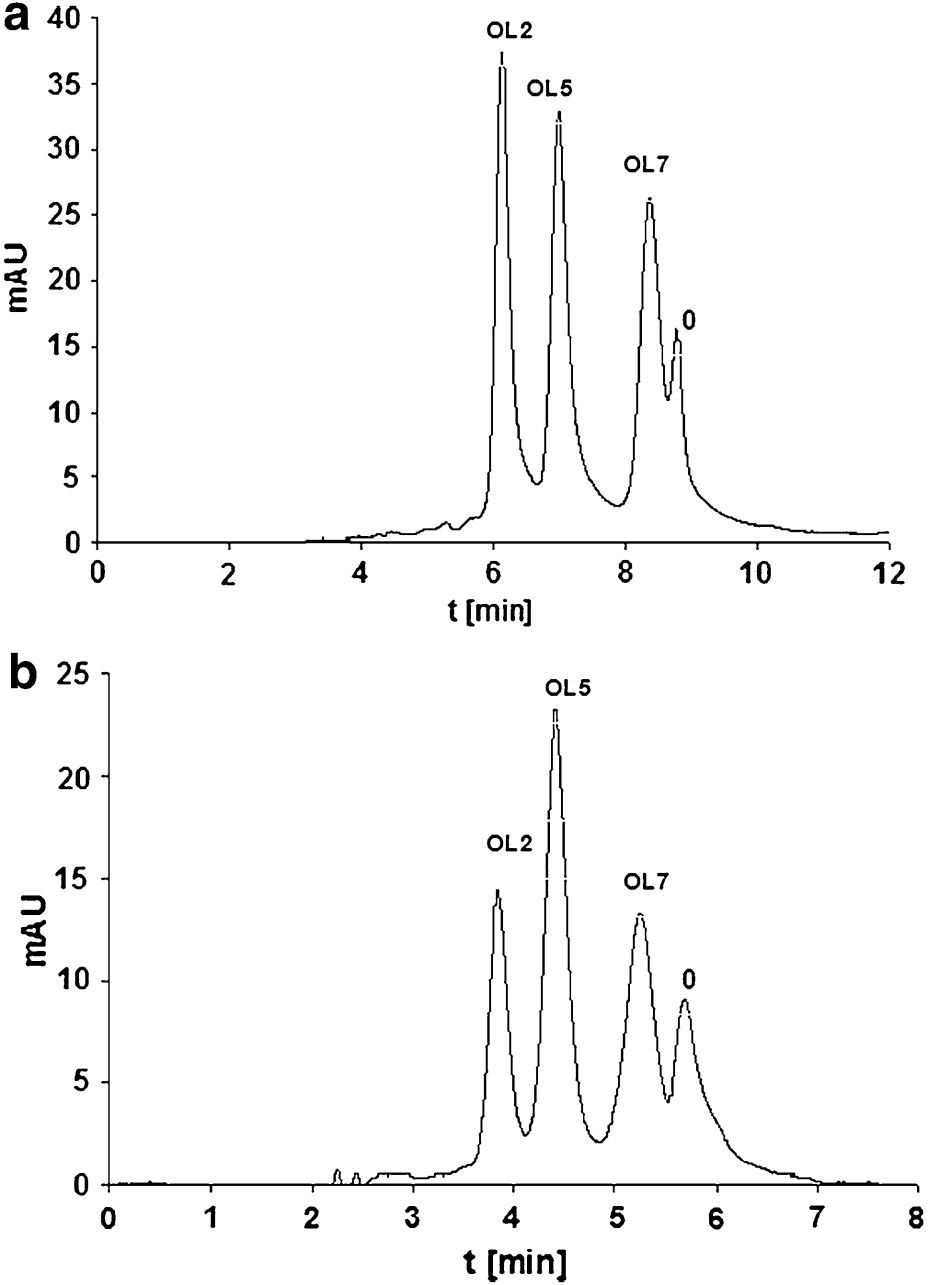
Chromatograms of separation of three-component mixture. Chromatographic conditions: (**a**) SG-AP, isocratic elution: 90 % (v/v) 100 mM TEAA, 10 % (v/v) ACN, flow rate 0.8 mL min^−1^;(**b**) SG-CHOL, isocratic elution: 87 % (v/v) 100 mM TEAA, 13 % (v/v) ACN, flow rate 1.0 mL min^−1^. The autosampler and column temperature 30 °C. UV–Vis detection λ = 254 nm. Injection volume 1.0 µL. Abbreviations of oligonucleotides names may be found in the "Materials and Methods" section and in Table 1. The peak 0—impurity

Although none of the tested stationary phases allowed for complete separation of all eight oligonucleotides, resolutions of three- and four-component mixtures were satisfactory. The exemplary chromatograms obtained by use of SG-AP and SG-CHOL are presented in Fig. 2. Both analyses were done with mobile phase containing TEAA buffer under isocratic elution mode, while utilization of octadecyl packing material requires developing of suitable gradient. The chromatogram for SG-AP, shown in Fig. 2a, illustrates the separation of ternary mixture in the isocratic mode with 10 % (v/v) of 100 mM TEAA and 90 % (v/v) of acetonitrile. The use 0.8 mL min^−1^ flow rate shortened separation time to 10 min. The chromatogram for SG-CHOL is presented in Fig. 2b. In this case, the mobile phase was composed of 13 % (v/v) 100 mM TEAA and 87 % (v/v) acetonitrile. Separation time was less than 7 min.

The resolution factor (*R*_*s*_) had value higher than 1.2 for SG-AP (Fig. 2a) and 1.8 for SG-CHOL (Fig. 2b). The best values of asymmetry factor were obtained for SG-CHOL (*f*_AS_ = 0.94–1.28). In contrast, the most asymmetrical peaks were obtained by use of SG-AP (*f*_AS_ = 0.93–1.52). This was caused by the influence of the polar groups present in the structure of alkylamide packing material. Interesting is the presence of an additional peak in all of the chromatograms. Oligonucleotides were purified by desalting. Therefore, with high probability, this additional signal was from impurities present in the sample after the synthesis process. This observation confirms that ion-pair chromatography is a useful tool in determining the purity of synthetic oligonucleotides.

## Concluding Remarks

The present study has shown that cholesterol and alkylamide stationary phases can be useful tool in ion-pair chromatography mode under non-denaturing conditions. Application of these packing materials allows separation of semi-complementary oligonucleotides of the same length, but bearing a single base substitution. It was achieved under non-denaturating conditions at low temperature of 30 °C and neutral pH. Despite self-complementary structure and formation of hairpin loops, studied oligonucleotides were separated. It proved utility of cholesterol and alkylamide stationary phases in the separation of oligonucleotides. Application of SG-AP and SG-CHOL resulted in shorter analysis time, but provided similar resolution of oligonucleotides in comparison with the SG-C18.

The selectivity factor was higher for pairs of oligonucleotides differing in the type of terminal nucleotides in comparison with pairs of various internal positions of nucleobases. It may be concluded that the access of 3′- or 5′-end of oligonucleotide to the stationary phase surface is greater in comparison with large hairpin loop. Consequently, the greater retention differences between analytes of various terminal nucleotide positions were observed.

In addition, it was confirmed that oligonucleotide retention is determined by the type and concentrations of IP reagents. The retention time of the studied compounds increased with an increase in alkylammonium salts hydrophobicity and concentration. Finally, it was also demonstrated, that mobile phases containing TEAA buffer have higher separation efficiency in comparison with HFIP/TEAA for the analysis of tested biomolecules.
